# Eugenol Improves Follicular Survival and Development During *in vitro* Culture of Goat Ovarian Tissue

**DOI:** 10.3389/fvets.2022.822367

**Published:** 2022-04-28

**Authors:** R. F. Silva, L. F. Lima, Anna C. A. Ferreira, A. F. B. Silva, D. R. Alves, B. G. Alves, A. C. Oliveira, Selene M. Morais, Ana Paula R. Rodrigues, Regiane R. Santos, J. R. Figueiredo

**Affiliations:** ^1^Laboratory of Manipulation of Oocytes and Preantral Follicles, Faculty of Veterinary, State University of Ceara, Fortaleza, Brazil; ^2^Natural Product Chemistry Laboratory, State University of Ceara, Fortaleza, Brazil; ^3^Superior Institute of Biomedical Science, State University of Ceará, Fortaleza, Brazil; ^4^Schothorst Feed Research, Lelystad, Netherlands

**Keywords:** goat, *in-vitro* culture, eugenol, antioxidant, preantral follicles

## Abstract

This study evaluated the effects of different concentrations (10, 20, or 40 μM) of eugenol (EUG 10, EUG 20, or EUG 40), ascorbic acid (50 μg/mL; AA) or anethole (300 μg/mL; ANE 300) on the *in-vitro* survival and development of goat preantral follicles and oxidative stress in the cultured ovarian tissue. Ovarian fragments from five goats were cultured for 1 or 7 days in Alpha Minimum Essential Medium (α-MEM+) supplemented or not with AA, ANE 300, EUG 10, EUG 20 or EUG 40. On day 7 of culture, when compared to MEM, the addition of EUG 40 had increased the rate of follicular development, as observed by a decrease in the proportion of primordial follicles alongside with an increase in the rate of normally developing follicles. Furthermore, EUG 40 significantly increased both follicular and oocyte diameters. Subsequently, ovarian fragments from three goats were cultured for 1 or 7 days in α-MEM+ supplemented or not with AA, ANE 300 or EUG 40. All tested antioxidants, except ANE 300, were able to significantly decrease the levels of reactive oxygen species in the ovarian tissue, but EUG 40 could most efficiently neutralize free radicals. All ovarian tissues cultured in the presence of antioxidants, especially EUG 40, presented a significant decrease in H3K4me3 labeling, indicating a silencing of genes that play a role in the inhibition of follicular activation and apoptosis induction. When compared to cultured control tissues, both EUG 40 and ANE 300 significantly increased the intensity of calreticulin labeling in growing follicles. The mRNA relative expression of ERP29 and KDM3A was significantly increased when the culture medium was supplemented with EUG 40, indicating a response to ER stress experienced during culture. In conclusion, EUG 40 improved *in-vitro* follicle survival, activation and development and decreased ROS production, ER stress and histone lysine methylation in goat ovarian tissue.

## Introduction

The mammalian ovary contains thousands to millions of immature oocytes, of which most (circa 90%) are enclosed in ovarian preantral follicles (PAFs—primordial, primary and secondary ones). Despite this large oocyte population, only around 0.1% will be ovulated; the remaining ones will suffer atresia (1). To safeguard part of the follicular population before degeneration, the *in-vitro* culture of PAFs has been developed. Such a procedure supports the preservation of genetic material from the farm and endangered animals (2, 3), the treatment of infertility in humans (4) and for the testing of chemical compounds (3, 5–7). Preantral follicles can be cultured *in situ* (enclosed in ovarian tissue) or in isolated form (8). *In-situ* culture aims to study the initial development of primordial and primary follicles (2, 8), whereas the culture of isolated follicles can be applied for the study of secondary follicles (8).

*In-vitro* culture of PAFs enclosed in the ovarian tissue may result in remarkable follicular losses (50–70%), which may be related to the high production of reactive oxygen species (ROS), leading to oxidative stress (9, 10). The ROS are substances normally produced through cellular metabolism and some cellular processes such as oocyte maturation (11), follicular steroidogenesis and ovulation (12). However, in stressful situations, such as under *in-vitro* culture conditions, the exceeding ROS can cause DNA damage, peroxidation of membrane phospholipids and cell death (13, 14). Oxidative stress will interfere with protein folding, which can be assessed by the activation of chaperones such as calreticulin and endoplasmic reticulum (ER) (15). Epigenetically, the methylation of histone residues plays an important role during folliculogenesis. For instance, oxidative stress interferes with histone methylation (16, 17), and such an effect is also observed during the *in-vitro* maturation of oocytes (18). The methylation of the histone 3 (H3), such as the methylation of H3K4me3 and H3K9me3, is associated with the activation and the silencing of gene expression, respectively (19, 20), and their demethylation is controlled by the lysine demethylases KDM1AX1 and KDM3A.

To avoid the negative impacts of ROS, antioxidants are added to the culture media. These antioxidants are classified as enzymatic when they are produced by cells (e.g., glutathione) or as non-enzymatic when they are obtained via the diet (e.g., ascorbic acid, anethole). Both ascorbic acid and anethole have previously been tested in the *in-vitro* culture of PAFs, resulting in a survival rate ranging from 40 to 60% and in follicular activation of around 65% (9, 10). A promising antioxidant that may increase the rate of viable follicles during *in-vitro* culture is the phenolic compound eugenol, due to its positive results obtained with other cell types (20, 21).

Eugenol is a phenolic compound, chemically designated as 4-allyl-2-methoxy-phenol, commonly known as clove essence because it is present in large quantities in clove essential oil (*Eugenia aromatica*), which has antimicrobial, anti-inflammatory, antiseptic, antipyretic and antioxidant properties (22). As an antioxidant, eugenol added to the *in-vitro* culture medium improves the viability (90%) of neuronal stem cells (23) and increases the expression of antioxidant enzymes (reduced glutathione, peroxidase, catalase and superoxide dismutase) in human mononuclear cells (20). Furthermore, eugenol is chemically stable during culture, with direct and indirect effects against ROS (24). Based on a previous study, eugenol supports the viability of bovine large secondary follicles *in vitro* (25). However, to the best of our knowledge, the effect of eugenol on *in-vitro* early folliculogenesis, i.e., survival and activation of the primordial follicle and initial growth of these follicles, is still unknown.

Although *in-vitro* culture of preantral follicles is a promising alternative to understand early folliculogenesis, oxidative stress during culture remains a challenge (9, 10). Based on this, the present study evaluated the effects of adding different concentrations of eugenol on the *in-vitro* development of goat preantral follicles. Additionally, *in-vitro* culture was also performed in the presence of the antioxidants commonly added to the medium for the culture of preantral follicles, i.e., anethole 300 μg/mL and ascorbic acid 50 μg/mL. The following endpoints were evaluated: follicular survival, activation, viability methylation profile (H3K4me3 and Calreticulin), gene expression (CAT; ERP29; KDM1AX1 and KDM3A) and antioxidant capacity.

## Materials and Methods

### Chemicals and Media

Unless otherwise mentioned, the culture media, ascorbic acid, anethole, eugenol and other chemicals used in the present experiments were purchased from Sigma-Aldrich (USA).

### Source of Ovaries

All procedures were approved by the Ethics and Animal Use Committee of State University of Ceara, Brazil (Protocol N. 05498214/2019). A total of eight ovarian pairs from eight adult crossbred goats (1–3 years old) were collected at a local slaughterhouse. The surrounding fat tissue and ligaments were removed, and the ovaries were washed in 70% alcohol, followed by two washes in minimum essential medium (MEM) plus HEPES (MEM-HEPES). The ovaries were placed into tubes containing 15 mL of MEM-HEPES, supplemented with penicillin (100 μg/mL) and streptomycin (100 μg/mL) and then transported to the laboratory at 4°C within 1 h.

### Culture Medium

The basic medium used was α-MEM^+^ (M5650, pH 7.2 −7.4) supplemented with 1.25 mg/mL bovine serum albumin (BSA), ITS (10 μg/mL insulin, 5.5 μg/mL transferrin, 5 ng/mL selenium), 2 mM glutamine, and 2 mM hypoxanthine, which was also named α-MEM^+^. Incubation was carried out at 38.5 °C in 7.5% CO_2_ in air for 1 or 7 days. Fresh media were prepared immediately before use and incubated for 1 h prior to use, with 1 mL in each well. The fragments were individually cultured in a 24-well plate containing 1 mL α-MEM^+^ culture medium. The culture medium was replaced with fresh medium every 2 days. Each treatment was replicated five times.

### Experimental Design

In the laboratory, the ovarian cortex of five ovarian pairs was removed with a sterile scalpel and divided into 14 fragments (3 x 3 x 1 mm). Two fragments were randomly taken and immediately fixed for the non-cultured control treatment as described below. The remaining fragments were randomly distributed (two fragments per treatment) into the six treatments as follows: α-MEM+ (cultured control treatment) either or not supplemented with ascorbic acid 50 μg/mL (AA treatment), anethole 300 μg/mL (ANE 300 treatment), or eugenol at three different concentrations of 10, 20 and 40 μM, which were termed as EUG 10, EUG 20, EUG 40 treatments, respectively. The concentration of ascorbic acid (9), anethole (10), and eugenol (10 μM/mL, 20 μM /mL or 40 μM/mL; 25) were chosen based on previous studies. At the end of each culture period (days 1 and 7), ovarian fragments from each treatment were evaluated for morphology and development (follicular class and growth of follicle and oocyte diameters). In addition, spent culture media at the end of 24 h of culture at day 1 and at day 7 of culture (after medium replacement at day 6) were stored at −80°C for ROS analysis and quantification of thiol groups.

Based on the results of the morphologic analysis, the viability, immunofluorescence detection of histone H3 methylated and calreticulin assays were performed only in the treatments with the concentration of eugenol that maintained follicular morphology and increased follicular activation and diameter. For this, three ovarian pairs collected at a slaughterhouse were prepared in the laboratory as aforementioned and then cut into fragments. One fragment was immediately processed for follicle isolation (fresh control) or fixed, and the remaining fragments were cultured for 7 d in basic culture medium (α-MEM^+^) in the absence or presence AA, ANE 300 or in the eugenol treatment that provided the best outcome, i.e., a significantly higher percentage of morphologically normal follicles at day 7 of culture when compared with the other treatments.

### Morphological Analysis and Assessment of *in vitro* Follicular Growth

Tissues from all treatments (fresh control or cultured for 1 and 7 days) were fixed in Carnoy's solution for 12 h and were processed for histology. The follicle stage and survival were assessed microscopically on serial sections. Coded anonymized slides were then examined by microscopy (Nikon, Sendai, Japan) at 400 × magnification.

The developmental stages of follicles have been defined as primordial (oocyte surrounded by a few flattened granulosa cells) or developing, i.e., intermediate (oocyte surrounded by flattened and at least one cuboidal granulosa cell), primary (oocyte surrounded by a complete layer of cuboidal granulosa cells), or secondary (oocyte surrounded by two or more complete layers of cuboidal granulosa cells). These follicles were classified as morphologically normal (follicles containing an intact oocyte and granulosa cells well-organized in layers without a pyknotic nucleus) and degenerated follicles (oocyte with a pyknotic nucleus, retracted cytoplasm, or disorganized granulosa cells detached from the basement membrane), as described Silva et al. (26).

Overall, 150 follicles were evaluated for each treatment (30 follicles per treatment in one repetition x 5 repetitions). To calculate follicular activation and growth, only morphologically normal follicles with a visible oocyte nucleus were recorded, and the proportion of primordial and growing follicles was calculated at day 0 and after day 7 of the culture in all treatments. In addition, from the basement membrane, the diameter of each normal oocyte and follicle were measured using a light microscope fitted with an eyepiece micrometer (Zeiss, Cologne, Germany) under 400 × magnification.

### Assessment of Preantral Follicle Viability by Trypan Blue

Each ovarian pair of ovaries (*n* = 3) collected at a slaughterhouse was cut into fragments. Two fragments were immediately processed for follicle isolation (non-cultured tissue), and the remaining fragments were cultured for 7 days in basic culture medium (α-MEM+), and ANE 300 or in the treatment group (40 μM/mL eugenol treatment) that provided the best outcome, i.e., a significantly higher percentage of morphologically normal follicles at day 7 of culture when compared with the other treatments. Goat preantral follicles were isolated from ovarian fragments using the mechanical method and stained with trypan blue as previously described (27). The viability of the isolated preantral follicles was assessed by the trypan blue dye exclusion test. The follicles were examined with an inverted microscope (Nikon, Tokyo, Japan) and classified as nonviable or viable if they were positively or negatively stained with trypan blue, respectively.

### Immunofluorescence Detection of Histone H3 Methylated and Calreticulin

The ovarian fragments were fixed with 4% paraformaldehyde in PBS (pH 7.2) for 4 h and later dehydrated, diaphanized, and included in paraffin. Briefly, tissue sections (5 μm) were mounted on Superfrost Plus slides (KnittelGlass, Bielefeld, Germany), dewaxed with Citrisolve (Fisher Scientific, Ottawa, Canada), and rehydrated with increasing ethanol concentration. Antigen recovery was performed by incubating tissue sections in 0.01 M sodium citrate buffer (pH 6) for 5 min under a pressure cooker. After cooling to 37°C, the tissue sections were washed in PBS and blocked for 1 h at room temperature using PBS containing 1% (w/v) BSA. After antigenic recovery, the slides were incubated overnight at 4°C with polyclonal primary antibodies H3K4me3 (Abcam ab8580) and calreticulin (Abcam ab2907). The negative control was obtained by omitting the primary antibodies. Subsequently, the slides were incubated with secondary IgG antibody labeled (Alexa Fluor^®^ 488; Abcam ab150077) for 1 h at room temperature and mounted with Fluoroshield Mounting Medium With DAPI^®^ (Abcam ab104139). Finally, the slides were viewed using a fluorescence microscope (Nikon, Tokyo, Japan) with 20 × magnification. The images were individually saved using a monochrome digital camera to measure fluorescence intensities (FIs). Exposure gains and rates were consistent across samples. The FIs were quantified only in the limited area of normal (*n* = 230) preantral follicles using the software Image J 1.4.7 (NIH, Bethesda, MD, US). All evaluations were performed by a single operator.

### RNA Extraction and Real-Time PCR (QPCR)

Total RNA was isolated from frozen-thawed samples (non-cultured and cultured) using the Trizol^®^ reagent, according to the manufacturer's recommendations (Invitrogen, Carlsbad, CA, USA). Isolated RNA was further purified (PureLink™ RNA Mini Kit; Ambion^®^, Carlsbad, CA, USA), treated with DNase I, and RNA samples were quantified with a spectrophotometer. Isolated RNA served as a template to synthesize the complementary DNA using the Superscript™ II RNase H-reverse Transcriptase (Invitrogen, São Paulo, SP, Brazil), followed by quantitative polymerase chain reaction (qPCR) in a final volume of 20 μL containing 1 μL of each complementary DNA, one x Power SYBR Green PCR Master Mix (10 μL), 7.4 μL of ultra-purewater, and 0.5 μL (final concentration) of both sense and antisense primers. Primer pairs (sense and antisense) and sequences for target genes are shown in Table 1. Peptidylprolyl isomerase A (PPIA) gene was selected as the endogenous control for normalizing gene expression among samples. The PCR conditions consisted of an initial step for denaturation and polymerase activation for 15 min at 94°C, followed by 40 cycles of 15 s at 94°C, 30 s at 60°C, and 45 s at 72°C. A final extension was performed for 10 min at 72°C. The specificity of each primer set was tested with melting curves that were carried out between 60°C and 95°C. All amplifications were carried out in a Bio-Rad iQ5 (Bio-Rad, Hercules, CA). Data were analyzed using the efficiency-corrected Delta-Delta-Ct method (28). The fold-change values of the genes of interest were normalized using the geometric mean of the fold-change values of a housekeeping gene.

**Table 1:** Primer pairs used for Real-Time Reverse-Transcriptase PCR analysis.

**Target gene**	**Primer sequence (5' → 3')**	**Orientation**	**Genbank accession no**
*PPIA*	TCATTTGCACTGCCAAGACTG	Forward	XM_018047035.1
	TCATGCCCTCTTTCACTTTGC	Reverse	
*CAT*	AAGTTCTGCATCGCCACTCA	Forward	GI: 402693375
	GGGGCCCTACTGTCAGACTA	Reverse	
*ERP29*	CCTTCCCCTGGATACAATCACT	Forward	EU596595.1
	AGTTTTCAGCCAGACGCTTG	Reverse	
*KDM1AX1*	ATGGGATTCGGCAACCTCAA	Forward	XM_005676879.3
	GCATCGGCCAACAATCACAT	Reverse	
*KDM3A*	TGCGTGGTTGGACAGGTTAA	Forward	XM_018055359.1
	AGAACTGCACTGTTGGTCGT	Reverse	

### Evaluation of the Antioxidant Potential Through Concentration of Reactive Oxygen Species and Quantification of Thiol Groups in the Medium After *in vitro* Culture

For the quantification of ROS and thiol groups, 60 μL of each spent culture medium was centrifuged (720 × g, 4°C) for 10 min to eliminate cells and cell debris before quantification. The supernatant was used for the analyses.

The concentration of ROS was assessed through the Amplex Red/HRP (Molecular Probes) method which detects the clotting of fluorescent oxidized substances in the reacting mixture. 10 μL of the medium were incubated with SOD (SOD 100 IU/mL; Sigma-Aldrich, St. Louis, MO, USA), the horseradish peroxidase (PRF 0.5 U/ml; Sigma-Aldrich, St. Louis, MO, USA) and AmplexRed (50 M; Sigma-Aldrich, St. Louis, MO, USA). The fluorescence was measured through a microdishes reader (Victor3, Perkin Elmer) at 595 nm of excitation. The levels of ROS were expressed in micromoles of hydrogen peroxide (H2O2).

For the analysis of thiol groups, 60 μL the supernatant of the centrifuged medium was added mixed with 930 μL of 10 mM Tris-HCl, 0.9% NaCl (w/v), pH 7.4 buffer and methanol with an ultra-turrax homogenizer (Jamke & Kunkel IKA Labortechnik, Germany). The quantification of free thiol groups in the medium was performed using 10 μL of dithiolnitrobenzoic acid (DTNB) in a spectrophotometer (Hitachi U-3300). Thiol groups react with DTNB releasing the disulfide joined to the molecule, leading to the formation of 2-nitro-5-benzoato (NTB-) which ionizes the c in aqueous medium with neutral or alkaline pH. The NTB2- is quantified with spectrophotometer at a wavelength of 412 nm.

### Evaluation of Antioxidant Capacity by Reducing ABTS and DPPH Free Radicals in the Medium After *in vitro* Culture

Antioxidant activity was measured in 96-well flat-bottom plates using BIOTEK Elisa reader (model ELX 800, software “Gen5 V2.04.11”). For the ABTS method, ABTS^+^ solution (7 mM, 5 mL) was mixed with 88 μL of potassium persulfate (140 mM). The mixture was stirred and kept in the dark at room temperature for 16 h. Then, 1 mL of this solution was added to 99 mL of ethanol. In 96-well plates, 300 μL of ABTS solution and 3 μL of the culture medium (sample) were used per well. For the DPPH analysis, the following solutions were used per well: 180 μL of DPPH (2,2-diphenyl-1-picrylhydrazyl) methanolic solution and 20 μL of the culture medium (sample).

The dilutions of samples and positive standards used in the quantitative microplate evaluations, starting from a stock solution with a concentration of 20 mg/mL were: 200 μg/mL, 100 μg/mL, 50 μg/mL, 25 μg/mL, 12.5 μg/mL, 6.25 μg/mL, 3.12 μg/mL, 1.56 μg/mL, and 0.78 μg/mL.

The absorbance for the ABTS method was measured at 630 nm up to a total of 10 min of incubation. As a negative standard, all solutions except the sample were used. For DPPH, the absorbance was measured at 490 nm for a total of 60 min of incubation.

The results were expressed as a percentage of inhibition and calculated according to the following formula:


$$\begin{array}{l} PI\%=\;\frac{AC-AS\;}{AC}\;\times 100 \end{array}$$

AC: Absorbance of the ABTS or DPPH control at time 0.
AS: Absorbance of the sample containing ABTS at time 6 and 10 min or DPPH at time 60 min.

### Statistical Analysis

Statistical analysis was carried out using Sigma Plot 11 (Systat Software Inc., USA). The normality (Shapiro-Wilk test) and homogeneity of variance (Levene's test) were initially evaluated. All parameters were analyzed with one-way ANOVA followed by Kruskal-Wallis and Mann-Whitney tests, when appropriate. Data are reported as mean (±SEM). Values with *P* < 0.05 were considered statistically significant.

## Results

### Follicle Survival Before and After Culture

**Figure 1:**
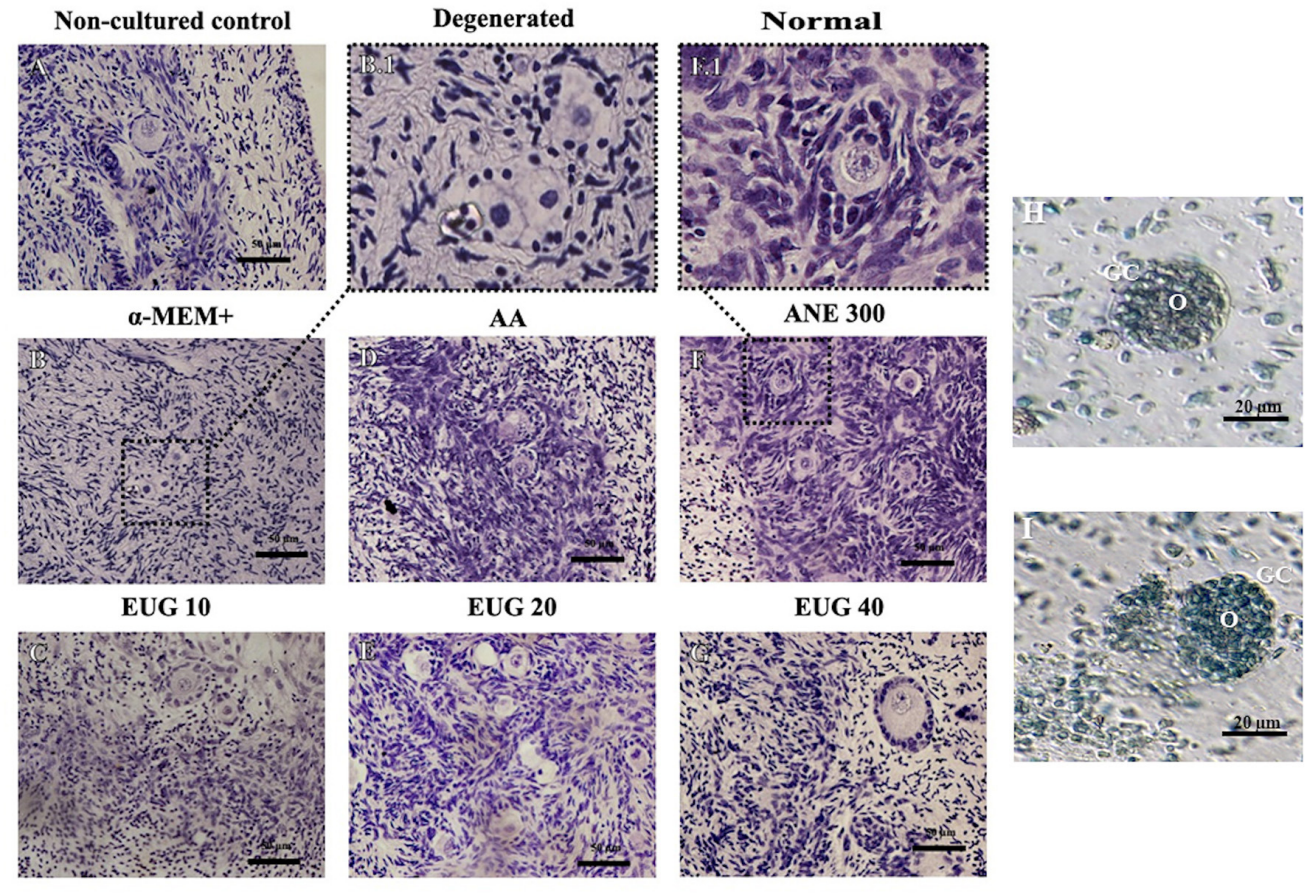
Representative images showing histological aspects of morphological normal preantral follicles before culture **(A)** and after culture in the absence of antioxidants **(B)**, degenerated follicles when cultured in the absence of antioxidants **(B.1)**, and normal follicles when cultured in a medium supplemented with EUG 10 **(C)**, or AA **(D)**, or EUG 20 **(E)**, or ANE 300 **(F,F.1)**, or EUG 40 **(G)**. Bar 50 μm. Staining: Periodic Acid-Schiff (PAS) and hematoxylin. Assessment of the viability of preantral follicles using trypan blue staining after 7-days culture. Viable **(H)** and non-viable **(I)** isolated preantral follicle after *in vitro* culture in α-MEM+. O, Oocyte; GC, Granulosa cells. Scale bar 20 μm.

A total of 2,632 preantral follicles were analyzed by classical histology. Representative images of the follicles after 7 days of culture are shown in Figure 1. The percentages of morphologically normal preantral follicles in each follicular category (primordial, intermediate and primary follicles) in the non-cultured control and after 1 or 7 days of *in-vitro* culture in all treatments are shown in Table 2. After 1-day culture, a significant reduction in the percentage of normal follicles was observed when compared to the control, except for primordial follicles cultured in the presence of EUG 20 or EUG 40, intermediate follicles cultured in the presence of ANE 300 or EUG 40 and growing follicles cultured in the presence of EUG 40. A significant decrease in the percentage of normal primary follicles was observed only when culture was performed for 1-day in the presence of ANE 300. On day-7 of *in-vitro* culture, except for the culture in the presence of ANE 300, a significant decrease in the percentage of normal primordial follicles was observed, whereas a decrease in the percentage of normal primary follicles was observed only when culture was performed in the presence of EUG 10. Although *in-vitro* culture decreased the morphology of growing follicles, the significantly highest percentages of normal growing follicles were observed after culture in the presence of EUG 40 and ANE 300. Moreover, a lower rate of normal primordial follicles was observed at day 7 when compared with day 1 when culture was performed in the presence of EUG, regardless of the tested concentration.

**Table 2:** Mean (±SEM) percentage of morphologically normal preantral follicles in each follicular category non-cultured ovarian tissue (control) and tissue cultured for 1 or 7 days in control medium without or with ascorbic acid 50 μg/mL (AA), anethole 300 μg/mL (ANE 300) or eugenol at 10 (EUG 10), 20 (EUG 20) or 40 μM/mL (EUG 40).

	**Control**	**α-MEM+**	**AA**	**ANE 300**	**EUG 10**	**EUG 20**	**EUG 40**
	**Day 0**	**Day 1**					
Primordial	85.9 ± 1.6^C^	74.5 ± 4.4^AB^	77.3 ± 1.7^B^	71.6 ± 4.9^A^	77.8 ± 2,7^B2^	83 ± 3.2^C2^	85.8 ± 3.1^C2^
Intermediate	100 ± 0.0^B^	70.9 ± 10.5^A^	61.6 ± 12.8^A^	74.5 ± 8.6^AB^	73.4 ± 16.9^A^	71.3 ± 21.5^A^	80 ± 44.7^AB^
Primary	100 ± 0.0^B^	87.5 ± 25^AB^	80 ± 44.7^AB^	59 ± 44.2^A^	66.6 ± 47.1^AB^	93.3 ± 14.9^AB^	100 ± 0.0^AB^
Growing	100 ± 0.0^B^	72.2 ± 10.7^A^	63.3 ± 10.5^A^	63.3 ± 20.3^A^	73.1 ± 16.9^A^	75.4 ± 15^A^	93.3 ± 14.9^B^
	**Day 0**	**Day 7**					
Primordial	85.9 ± 1.6^C^	57.7 ± 15.4^A^	63.7 ± 14.9^AB^	79.9 ± 19.5^BC^	52.6 ± 6.3^A1^	53.4 ± 11^A1^	60 ± 30.3^AB1^
Intermediate	100 ± 0.0^C^	74.8 ± 7.4^A^	70.1 ± 11.6^A^	77.7 ± 6^AB^	75.8 ± 11.5^A^	71.3 ± 14.1^A^	88.2 ± 6.7^B^
Primary	100 ± 0.0^B^	100 ± 0.0^AB^	NF	91.6 ± 14.4^AB^	50 ± 50^A^	83.3 ± 33.3^AB^	62.5 ± 47.8^AB^
Growing	100 ± 0.0^C^	75.9 ± 7.7^A^	70.1 ± 11.6^A^	77.9 ± 5.9^AB^	74.4 ± 11.3^A^	70.8 ± 14.5^A^	87.2 ± 5.1^B^

*^A−C^Different superscripts in a row indicate significant differences among treatments (P ≤ 0.05).*

*^1−2^Differs significantly between days of culture within the same treatments and follicular category (P ≤ 0.05).*

*NF, No Follicles were found*.

### Distribution Rates of Morphologically Normal and Degenerated Preantral Follicles (Primordial and Growing) Before and After *in vitro* Culture

**Figure 2:**
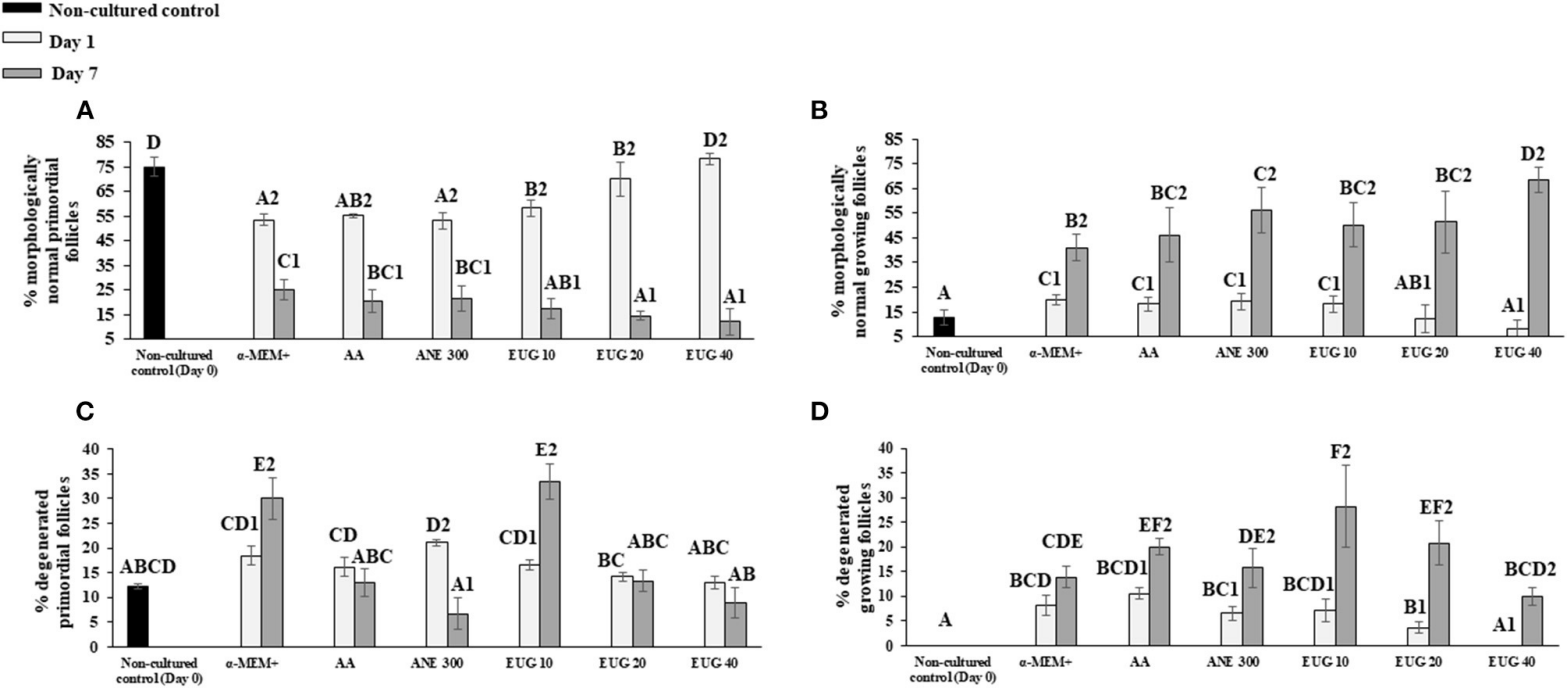
Mean (±SEM) percentage of normal **(A,B)** and degenerated **(C,D)** primordial and growing follicles in non-cultured ovarian tissue (control) and tissue cultured for 1 or 7 days in control medium with or without supplementation with ascorbic acid 50 μg/mL (AA), anethole 300 μg/mL (ANE 300), or eugenol at 10 (EUG 10), 20 (EUG 20) or 40 μM/mL (EUG 40). ^A−*F*^Differs significantly among treatments (*P* ≤ 0.05). ^1−2^Differs significantly between days of culture within the same treatments (*P* ≤ 0.05).

The proportions of morphologically normal primordial (Figure 2A) and growing follicles (intermediate + primary—Figure 2B) as well as degenerated primordial (Figure 2C) and growing (Figure 2D) follicles were determined in non-cultured ovarian tissue or in tissues cultured for 1 or 7 days. Regardless of the cultured treatment, from day 1 to day 7, there was a significant reduction in the percentage of normal primordial follicles, with a concomitant increase in the percentage of normally growing follicles. On days 1 and 7 of *in-vitro* culture, a decrease in the percentage of normal preantral follicles was observed in all treatments, except on day 1, when tissue was cultured in the presence of EUG 40. On day 7, medium supplementation with EUG 40 resulted in a decreased rate of normal primordial follicles together with the highest percentages of normally developing follicles.

### *In vitro* Growth of Preantral Follicles and Oocytes

The follicular and oocyte diameters in the non-cultured control treatment and after 1 or 7 days of culture are shown in Tables 3, 4, respectively. When compared to the non-cultured control, smaller follicular diameters were obtained when cultured in the presence of MEM alone (primordial—day 7, intermediate—days 1 and 7) or supplemented with AA (primordial and primary—day 7, and intermediate—days 1 and 7), ANE 300 and EUG 10 (primordial, intermediate and primary—day 7), EUG 20 (primordial, intermediate and primary—days 1 and 7) and EUG 40 (primordial—day 1). Moreover, the intermediate follicles from the EUG 40 presented a greater diameter than the non-cultured control group (Table 3). Regarding the oocyte diameter, regardless of the follicular category and the day of culture, the lowest diameters were obtained in groups grown *in vitro* in the absence or presence of antioxidants, except for the EUG 40 treatment. In this treatment, regardless of the follicular category, oocyte diameter was significantly higher than in the non-cultured control (Table 4). In general, in the tested treatments, a reduction in follicular and oocyte diameter was observed from day 1 to day 7 only in primordial and intermediate follicles. However, the EUG 40 treatment was the only treatment in which both follicular and oocyte diameters were increased significantly throughout the culture, regardless of the follicular category (Tables 3, 4). When compared to MEM, after 7 days of culture, except for the primordial follicle category in AA treatment, the addition of AA, ANE and EUG (regardless of the concentration) resulted in primordial, intermediate and primary follicles with significantly greater diameters. Concerning oocyte diameter, the addition of AA (only for intermediate follicle), ANE (primordial and intermediate follicles), EUG 10 and 20 (only for intermediate follicles) and EUG 40 (primordial, intermediate and primary follicles) significantly increased oocyte diameter after 7 days of culture. When the tested antioxidants were compared, except for the oocyte diameter of primary follicles, greater follicular and oocyte diameters were observed after 7 days of culture when EUG at 40 μM was added to the culture medium. Considering primary follicles, after 7 days of culture, a greater oocyte diameter was observed in the EUG 40 treatment when compared to the AA treatment (*P* < 0.05).

**Table 3:** Mean (±SEM) follicular in non-cultured ovarian tissue (control) and tissue cultured for 1 or 7 days in control medium without or with ascorbic acid 50 μg/mL (AA), anethole 300 μg/mL (ANE 300) or eugenol at 10 (EUG 10), 20 (EUG 20) or 40 μM/mL (EUG 40).

			**Follicular diameter (μm)**				
	**Control**	**α-MEM+**	**AA**	**ANE 300**	**EUG 10**	**EUG 20**	**EUG 40**
	**Day 0**	**Day 1**					
Primordial	35.5 ± 0.5^Ca^	34.5 ± 0.7^C2a^	33.8 ± 0.9^CB2a^	34.1 ± 0.7^C2a^	34.5 ± 0.6^C2a^	30.8 ± 0.8^A2a^	31.5 ± 0.8^AB1a^
Intermediate	42.5 ± 0.9^Cb^	38.2 ± 0.7^AB2b^	38.1 ± 0.7^ABb^	40.2 ± 0.7^BC2b^	41.2 ± 1.3^C2b^	37.3 ± 0.8^A2b^	40.2 ± 1.0^BC1b^
Primary	53.5 ± 2.0^Cc^	46.4 ± 1.3^AB2c^	47.3 ± 2.7^ABCc^	46.0 ± 1.4^ABCc^	51.0 ± 2.5^BC2c^	45.2 ± 1.5^Ac^	52.8 ± 1.5^C1c^
	**Day 0**	**Day 7**					
Primordial	35.5 ± 0.5^Ca^	26.8 ± 1.1^A1a^	28.6 ± 0.9^AB1a^	30.8 ± 0.8^B1a^	28.4 ± 0.9^A1a^	28.2 ± 0.9^A1a^	38.1 ± 0.6^C2a^
Intermediate	42.5 ± 0.9^Db^	29.8 ± 1.0^A1a^	38.0 ± 1.2^Cb^	37.3 ± 0.6^C1b^	33.5 ± 1.3^B1b^	33.5 ± 0.9^B1b^	49.1 ± 0.7^E2b^
Primary	53.5 ± 2.0^CBc^	52.4 ± 4.2^B1b^	43.1 ± 1.5^Ac^	43.5 ± 1.0^Ac^	44.4 ± 1.4^A1c^	44.1 ± 1.6^Ac^	60.6 ± 2.0^C2c^

*^A−E^Different superscripts in a row indicate significant differences among treatments (P ≤ 0.05).*

*^a−c^Different superscripts in a column indicate significant differences among treatments (P ≤ 0.05).*

*^1−2^Differs significantly between days of culture within the same treatments and follicular category (P ≤ 0.05)*.

**Table 4:** Mean (±SEM) oocyte diameter in non-cultured ovarian tissue (control) and tissue cultured for 1 or 7 days in control medium without or with ascorbic acid 50 μg/mL (AA), anethole 300 μg/mL (ANE 300) or eugenol at 10 (EUG 10), 20 (EUG 20) or 40 μM/mL (EUG 40).

			**Oocyte diameter (μm)**				
	**Control**	**α-MEM+**	**AA**	**ANE 300**	**EUG 10**	**EUG 20**	**EUG 40**
	**Day 0**	**Day 1**					
Primordial	26.4 ± 0.6^Ca^	25.4 ± 0.6^BC2a^	23.6 ± 0.6^AB2a^	23.6 ± 0.6^ABa^	23.3 ± 0.5^A2a^	23.3 ± 0.7^A2a^	22.1 ± 0.7^A1a^
Intermediate	30.4 ± 0.6^Cb^	26.5 ± 0.5^A2a^	26.8 ± 0.6^ABb^	27.4 ± 0.6^ABb^	27.8 ± 0.9^ABC2b^	28.4 ± 0.7^ABC2b^	28.9 ± 0.8^BC1b^
Primary	38.1 ± 1.5^Cc^	32.0 ± 1.0^ABb^	30.4 ± 1.5^Ac^	32.9 ± 1.1^ABc^	32.8 ± 1.4^ABc^	31.9 ± 2.1^ABc^	39.0 ± 1.3^C1bc^
	**Day 0**	**Day 7**					
Primordial	26.4 ± 0.6^Ca^	19.7 ± 1.0^A1a^	20.6 ± 0.7^AB1a^	21.7 ± 0.5^Ba^	20.2 ± 0.6^AB1a^	20.0 ± 0.7^AB1a^	28.8 ± 0.7^D2a^
Intermediate	30.4 ± 0.6^Db^	20.7 ± 0.9^A1a^	27.2 ± 0.8^Cb^	26.9 ± 0.6^Cb^	23.6 ± 1.0^B1b^	23.8 ± 0.6^B1b^	38.8 ± 0.7^E2b^
Primary	38.1 ± 1.5^Cc^	32.9 ± 2.3^ABb^	32.0 ± 1.6^Ac^	32.6 ± 1.3^ABc^	34.2 ± 1.6^ABCc^	33.8 ± 1.6^ABCc^	48.7 ± 1.8^B2c^

*^A−E^Different superscripts in a row indicate significant differences among treatments (P ≤ 0.05).*

*^a−c^Different superscripts in a column indicate significant differences among treatments (P ≤ 0.05).*

*^1−2^Differs significantly between days of culture within the same treatments and follicular category (P ≤ 0.05)*.

In the present study, the follicular and oocyte diameters were compared among the follicular categories, i.e., primordial, intermediate and primary follicles within the same treatment and day of culture (Table 3). Except for the MEM treatment, when similar (*P* < 0.05) follicular (day 7) and oocyte (days 1 and 7) diameters were observed between primordial and intermediate follicles, a significant and progressive increase in follicular and oocyte diameter was observed from the primordial to the primary follicle category both in the non-cultured control and in the cultured treated groups, regardless of the day of culture.

### Assessment of Follicle Viability After Culture

**Figure 3:**
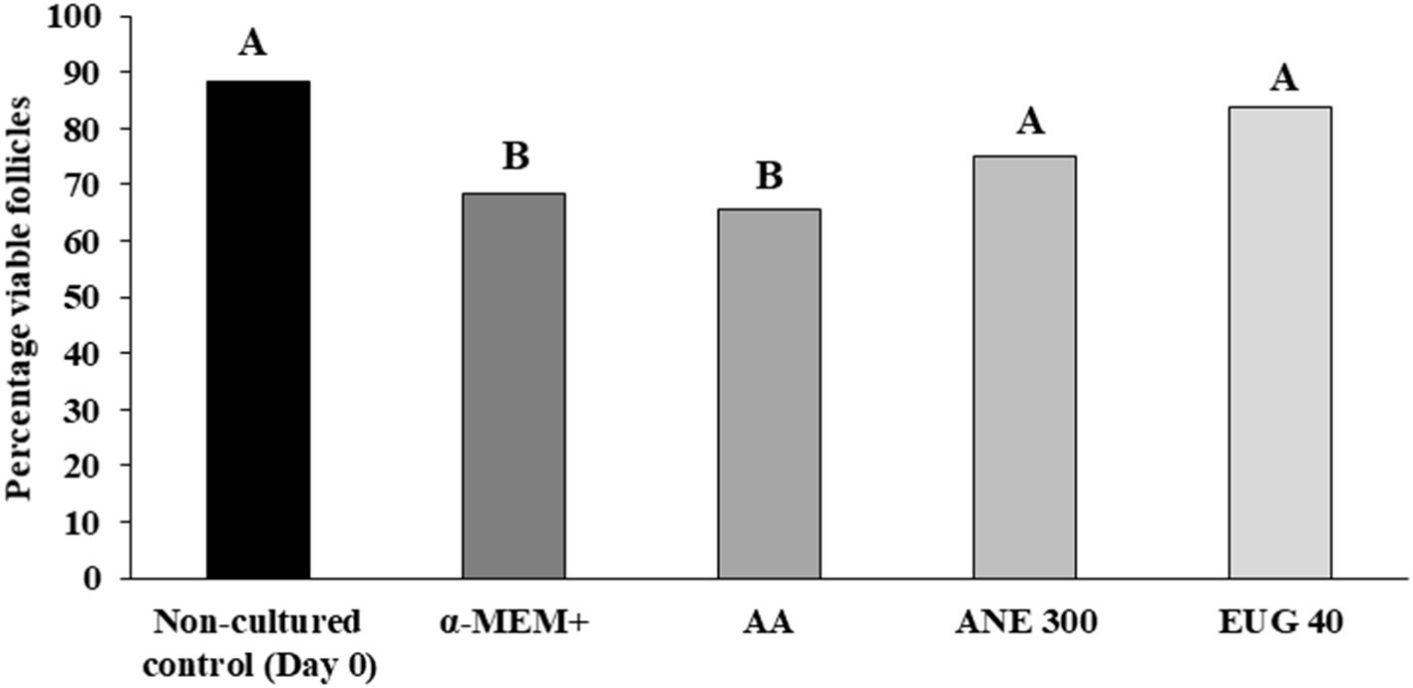
Percentages of viable goat preantral follicles in the non-cultured control group and after 7 days in control medium with or without ascorbic acid 50 μg/mL (AA), anethole 300 μg/mL (ANE 300) or eugenol 40 μM /mL (EUG 40). ^A, B^Different superscripts in a row indicate significant differences among treatments (*P* ≤ 0.05).

Follicles were classified as viable (Figure 1H) or non-viable (Figure 1I) if they were negatively or positively stained with trypan blue, respectively. After 7 days of culture, the percentages of viable follicles were similar among MEM (68.6%) ANE 300 (75.0%), AA (65.7%), and EUG 40 (83.9%) treatments. However, lower rates of viable follicles were observed in MEM and AA treatments than in the non-cultured control (88.2%) (Figure 3).

### Antioxidants Test

**Figure 4:**
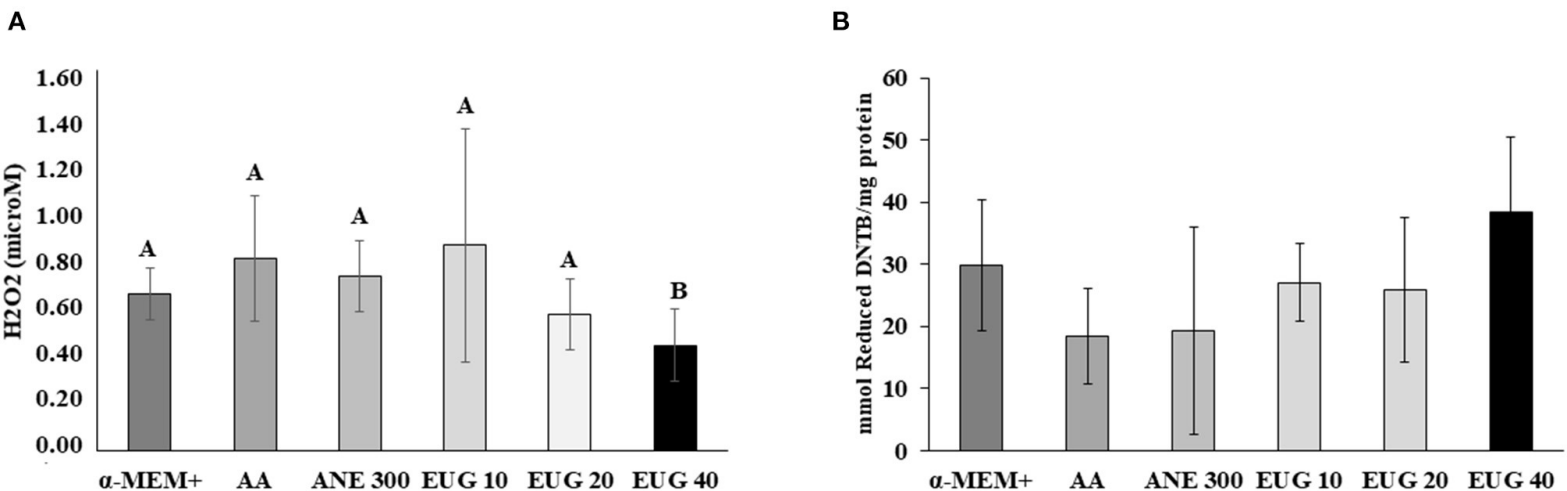
Mean (±SEM) levels of H2O2 production [microM; **(A)**] and reactive protein thiol (mmol reduced DTNB/mg protein) **(B)** in ovarian tissue cultured for 7 days. ^A, B^Different superscripts in a row indicate significant differences among treatments (*P* ≤ 0.05).

The quantification of H_2_O_2_ production and thiol groups was performed after 7 days of culture (Figure 4). In all treatments, H_2_O_2_ production was similar, except when EUG 40 was added to the medium, which resulted in a decrease in H_2_O_2_ production. No differences were observed when comparing thiol levels.

**Figure 5:**
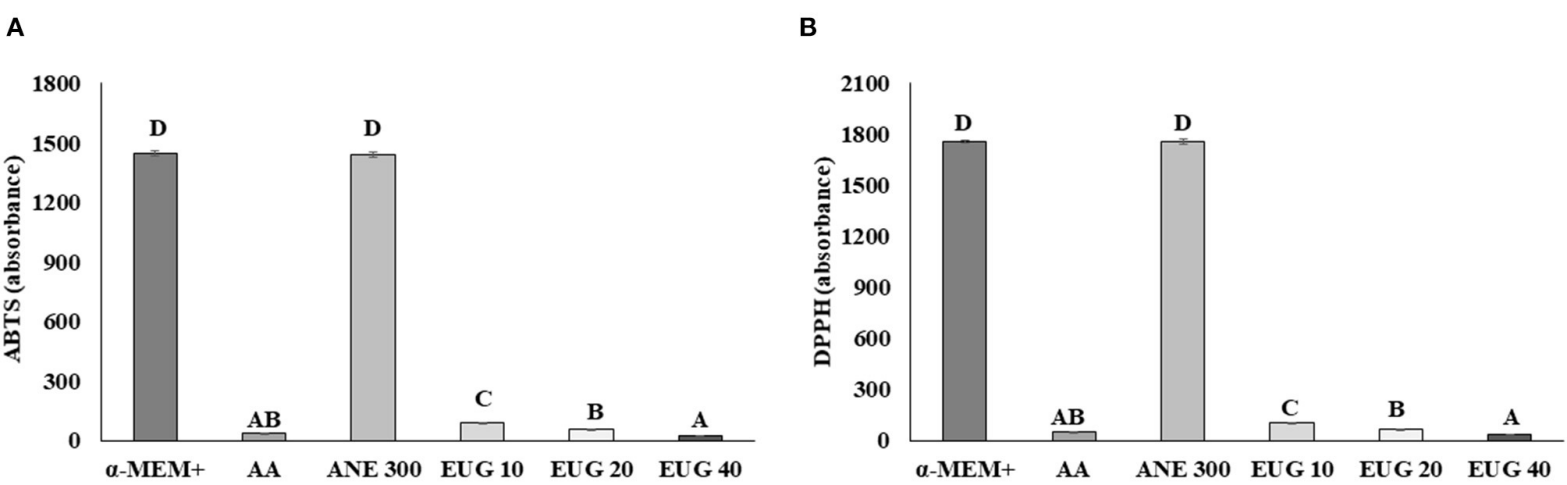
Mean (±SEM) reduction of ABTS **(A)** and DPPH free radical **(B)** levels after exposure to culture medium without or with supplementation with ascorbic acid 50 μg/mL (AA), anethole 300 μg/mL (ANE 300) or eugenol 40 μM /mL (EUG 40). Culture medium collected at day 7 of culture. ^A−*D*^Values with different superscripts differ among treatments (*P* ≤ 0.05).

The neutralizing capacity against ABTS and DPPH on day 7 is shown in Figure 5. All tested antioxidants, except ANE 300, were able to significantly decrease the levels of ABTS and DPPH when compared to the control. The highest capacity to neutralize free radicals was observed with EUG 40, whereas AA was similar to EUG 20 and 40.

### Immunofluorescence Detection of H3K4me3 and Calreticulin

**Figure 6:**
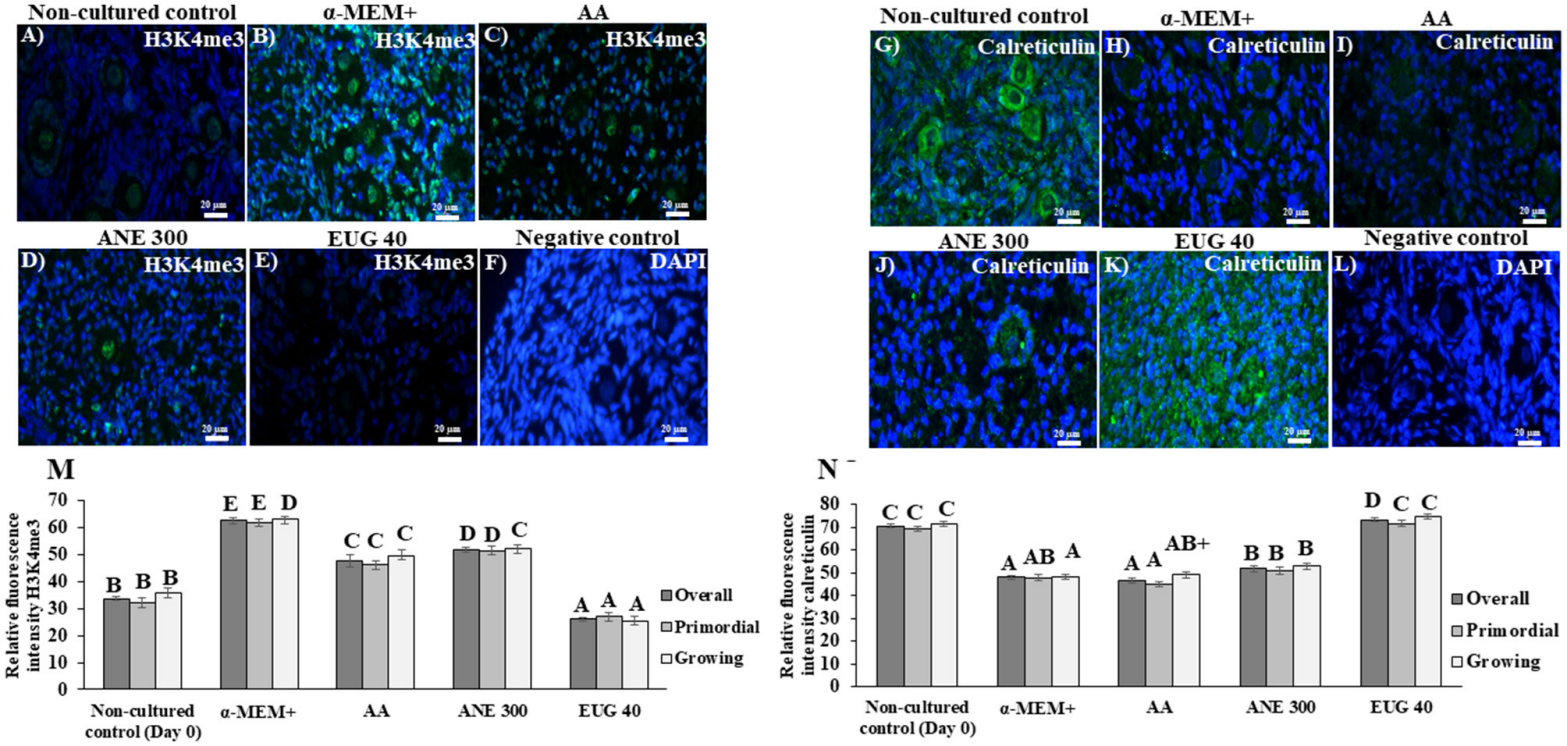
Immunofluorescence results showing the mean (±SEM) levels of H3K4me3 **(A–E)** and calreticulin **(G–K)**, negative control with DAPI **(F,L)**, quantification of H3K9me3 **(M)** and of calreticulin **(N)** signals in non-cultured ovarian tissue and tissue cultured for 7 days in medium without or with ascorbic acid 50 μg/mL (AA), anethole at 300 μg/mL (ANE 300) or eugenol 40 μM/mL (EUG 40). (Scale bar = 20 μm). ^A−*F*^Different superscripts indicate significant differences among treatments (*P* ≤ 0.05). ^+^Differs significantly between follicular category within in the same treatments (*P* ≤ 0.05).

Representative staining images of H3K4me3 (Figures 6A–F) and calreticulin (Figures 6G–L) follicles after 7 days of culture are shown. The labeling intensity of H3K4me3 after 7 days of *in-vitro* culture is shown in Figure 6M. All cultured tissues presented a similar intensity of H3K4me3 labeling, regardless of the follicular class, except when the ovarian tissue was cultured in the presence of EUG 40, resulting in a significant decrease of H3K4me3 labeling. All ovarian tissues cultured in the presence of antioxidants, especially EUG 40, presented a significant decrease in H3K4me3 labeling.

Figure 6N shows the labeling intensity for calreticulin after 7 days of *in-vitro* culture. Only culturing of primordial and growing follicles in the presence of EUG 40 resulted in a labeling intensity similar to that observed in the non-cultured tissue. When compared to cultured control tissues, both EUG 40 and ANE 300 significantly increased the labeling intensity of calreticulin in growing follicles. Such effect was observed in all follicular categories when EUG 40 was added to the culture medium.

### Relative Gene Expression

**Figure 7:**
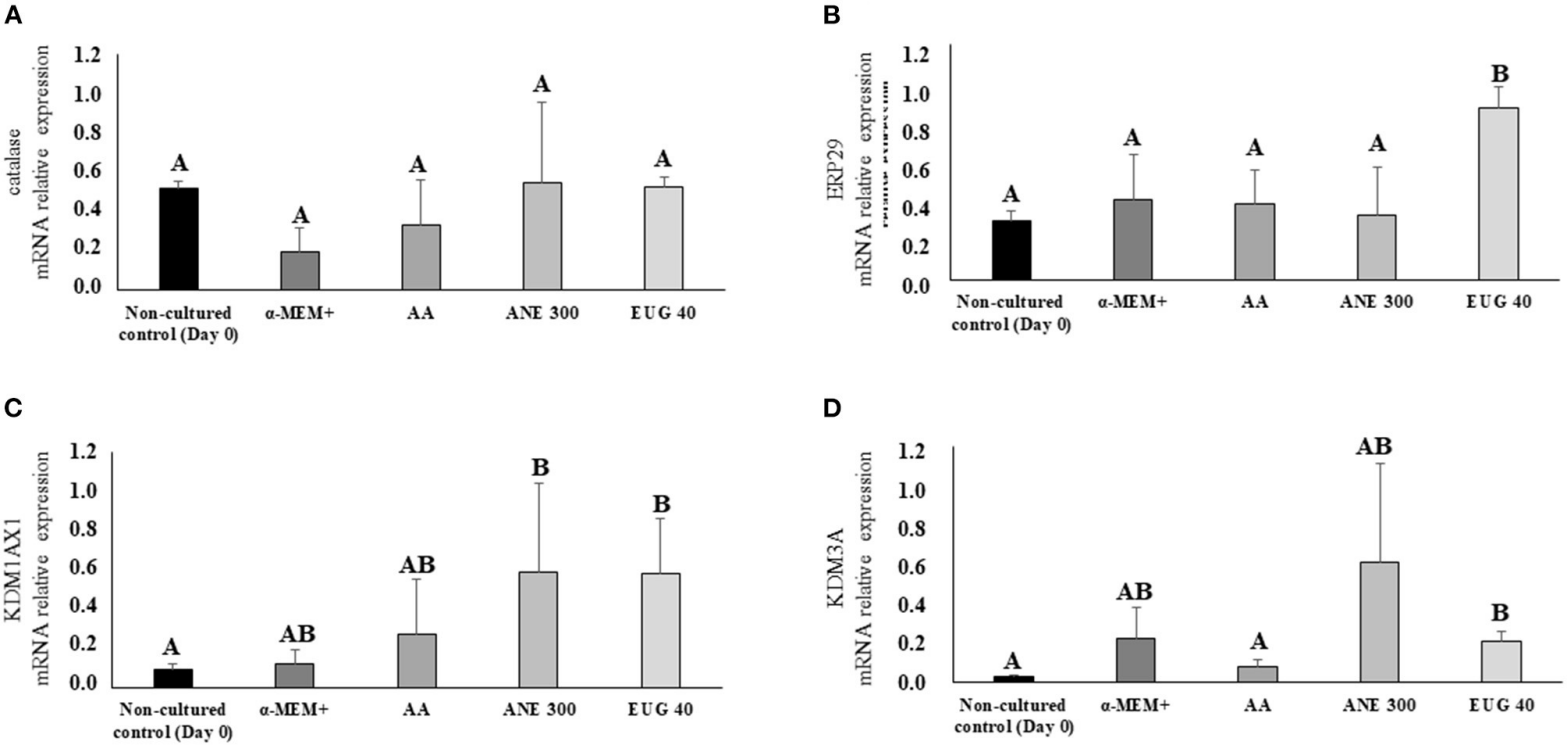
Mean (±SEM) relative mRNA expression of genes involved in cellular stress [CAT **(A)** and ERP29 **(B)**] and methylation [KDM1AX1 **(C)** and KDM3A **(D)**] in goat preantral follicles in the non-cultured control group and after 7 days in control medium without or with supplementation of 50 μg/mL of ascorbic acid (AA) or anethole at 300 μg/mL (ANE 300) or eugenol 40 ng/mL (EUG 40). ^A, B^Different superscripts indicate significant differences among treatments (*P* ≤ 0.05).

Figure 7 depicts the mRNA expression of the markers CAT (7A), ERP29 (7B), KDM1AX1 (7C) and KDM3A (7D). The mRNA expression of CAT was not affected by the treatments, whereas the mRNA relative expression of ERP29 and KDM3A was significantly increased when the culture medium was supplemented with EUG 40. The mRNA relative expression of KDM1AX1 was significantly increased when the culture medium was supplemented with EUG 40 or ANE 300.

## Discussion

The present study investigated the effects of eugenol (10, 20, and 40 uM), ascorbic acid (50 μg/mL) and anethole (300 μg/mL) on the *in-vitro* culture of caprine preantral follicles enclosed in ovarian tissue. In general, EUG 40 improved follicle survival, activation, and both follicular and oocyte growth and decreased ROS production and histone methylation in ovarian tissue after 7 days of culture.

During the first day of culture, only EUG 40 maintained follicular survival similar to non-culturedtissue, regardless of the follicular class, whereas ANE 300 maintained the morphology of intermediate follicles. Regardless of the treatment, 7-day *in-vitro* culture decreased the survival rates of the primordial, intermediate and growing follicles, except for ANE 300, which preserved the morphology of primordial and primary follicles. Only medium supplementation with EUG 40 or ANE 300 enhanced the survival rates of growing follicles. *In-vitro* culture of ovarian follicles enclosed in ovarian tissue requires a medium that supports the maintenance of the ovarian cortex, because stromal cells support the follicular development (29, 30). Sá et al. (10) demonstrated that anethole can support the maintenance of follicular morphology after 7 days of culturing by decreasing the ROS production. The antioxidant capacity of eugenol was confirmed by its ability to decrease the H_2_O_2_ production. Eugenol not only decreases the excess of ROS (24) but also enhances the activity of enzymatic antioxidants (25). Ighodaro and Akinloye (31) showed that eugenol mitigates the effects of H_2_O_2_ via the regulation of glutathione peroxidase levels. Furthermore, eugenol inhibits iron oxidation, regulating superoxide and hydroxyl levels (22, 23). In the present study, eugenol was able to decrease the levels of both ABTS and DPPH after a 7-day culture, indicating that this antioxidant has a long-term effect in culture. Chemically, eugenol is more stable and has a more long-term activity than anethole because of the presence of a –OH group in its aromatic ring (24). Such chemical stability is also found in ascorbic acid, and its antioxidant activity was previously compared with that of eugenol (32). However, when culturing ovarian tissue, we could not observe similar effects when comparing both antioxidants.

The mRNA relative expression of ERP29 and the increased expression of the protein calreticulin were observed in the tissue cultured in the presence of EUG 40, indicating that this antioxidant was protecting the follicles from ER stress. This was confirmed by a decrease in ROS production. The EPR29 regulates the connexin 43 in the Golgi complex, stabilizing these monomers (33). Connexins are membrane proteins that play a role in intercellular communication, allowing the transfer of ions, such as AMPc (34, 35). Tokuhiro et al. (36) demonstrated that calreticulin regulates the folding of the proteins Growth and Differentiation Factor 9 (GDF9) and Bone Morphogenetic Protein 15 (BMP15) in the ER. Both GDF9 and BMP15 are involved in the survival and growth of oocytes and follicles (17, 37, 38).

The addition of EUG 40 to the control medium improved follicular development after a 7-day culture. Besides the ability of eugenol to stimulate the production of antioxidant substances (glutathione peroxidase, superoxide dismutase and glutathione-S-transferase), this compound also enhances the synthesis of the protein zona occludens (39) and decreases the expression of interleukin-6 (IL-6) and Tumor Necrosis Factor-alpha (TNF-alpha) (21). The zona occludens proteins are present in developing follicles, favoring the antrum formation and the subsequent follicular development (36). Silva et al. (40) showed that during the initial folliculogenesis, TNF-α increases the number of apoptotic cells in cultured bovine ovarian tissue, and Manabe et al. (41) observed an impaired survival of primordial follicles.

The H3 is one of the constituents of the nucleosome protecting the DNA; the methylation of these proteins is associated with a decreased or increased transcription (42). In the present study, we evaluated the expression of markers for the two enzymes lysine demethylase, responsible for the removal of methyl radicals from the H3, KDM3A, and KDM1AX1. The KDM3A decreases gene silencing, including those related to the inhibition of primordial follicle activation and the induction of apoptosis (43–45). The relative mRNA expression levels for KDM3A and KDM1AX1 were increased in anethole and EUG 40 treatments when compared to the non-cultured control. However, EUG 40 was the only treatment that decreased H3K4me3 expression. Therefore, we suggest that the higher rates of follicle survival and activation observed in the EUG 40 may be explained by the silencing of pro-apoptotic genes as well as genes related to the maintenance of primordial follicle quiescence. To date, studies have shown that decreased H3K4me3 levels lead to a reduction in the expression levels of pro-apoptotic genes [Bax, Bad and Bid (44, 45)].

In conclusion, eugenol at 40 μM improved follicle survival, regardless of the follicular class, at day 1 of culture. At day 7, EUG 40 was the most efficient supplement to improve the survival of growing follicles and protected the ovarian tissue against ROS, ER stress as well as histone lysine methylation.

## Data Availability Statement

The original contributions presented in the study are included in the article/supplementary material, further inquiries can be directed to the corresponding author.

## Author Contributions

RSi, SM, AR, and JF: conceptualization. RSi, LL, AF, AS, DA, BA, and AO: methodology. RSi, RSa, and JF: validation and writing—original draft preparation. RSi, RSa, BA, and JF: formal analysis. RSi, LL, AF, AS, DA, and BA: investigation. AO, SM, AR, and JF: resources. RSi, RSa, AO, BA, and JF: writing—review and editing. RSi: project administration. JF: funding acquisition. All authors have read and agreed to the published version of the manuscript.

## Conflict of Interest

The authors declare that the research was conducted in the absence of any commercial or financial relationships that could be construed as a potential conflict of interest.

## Publisher's Note

All claims expressed in this article are solely those of the authors and do not necessarily represent those of their affiliated organizations, or those of the publisher, the editors and the reviewers. Any product that may be evaluated in this article, or claim that may be made by its manufacturer, is not guaranteed or endorsed by the publisher.

## References

[1] Van Der Hurk R Zhao J. Formation of mammalian oocytes and their growth, differentiation and maturation within ovarian follicles. Theriogenology. (2005) 63:1717–51. 10.1016/j.theriogenology.2004.08.005 15763114

[2] Figueiredo JR Rodrigues AP Silva JR Santos RR. Cryopreservation and *in vitro* culture of caprine preantral follicles. Reprod Fertil Dev. (2011) 23:40–7. 10.1071/RD10227 21366979

[3] Figueiredo JR Cadenas J Lima LF Santos RR. Advances *in vitro* folliculogenesis in domestic ruminants. Anim Reprod. (2019) 16:52–65. 10.21451/1984-3143-AR2018-0123 33936289 PMC8083813

[4] Picton HM Harris SE Muruvi W Chambers EL. The *in vitro* growth and maturation of follicles. Reproduction. (2008) 136:703–15. 10.1530/REP-08-0290 19074213

[5] Xu G Lin S Law WC Roy I Lin X Mei S. The invasion and reproductive toxicity of QDs-Transferrin bioconjugates on preantral follicles in vitro. Theranostics. (2012) 2:734–45. 10.7150/thno.4290 22916073 PMC3425092

[6] Silva TES de Brito DCC de Sá NAR da Silva RF Ferreira ACA da Silva JYG. Equol: a microbiota metabolite able to alleviate the negative effects of zearalenone during *in vitro* culture of ovine preantral follicles. Toxins. (2019) 11:652. 10.3390/toxins11110652 31717534 PMC6891317

[7] Brito DCC Silva IP Ferreira ACA Sá NAR Guedes MIF Rodrigues APR. Effects of in vitro exposure of sheep ovarian tissue to zearalenone and matairesinol on preantral follicles. Zygote. (2021) 25:1–4. 10.1017/S0967199421000794 34689852

[8] Telfer EE Binnie JP Mc Caffery FH Campbell BK. *In vitro* development of oocytes from porcine and bovine primary follicles. Mol Cell Endocrinol. (2000) 163:117–23. 10.1016/S0303-7207(00)00216-1 10963883

[9] Rossetto R Lima-Verde IB Matos MH Saraiva MV Martins FS Faustino LR. Interaction between ascorbic acid and follicle-stimulating hormone maintains follicular viability after long-term *in vitro* culture of caprine preantral follicles. Domest Anim Endocrinol. (2009) 37:112–23. 10.1016/j.domaniend.2009.04.003 19493642

[10] Sá NAR Bruno JB Guerreiro DD Cadenas J Alves BG Cibin FWS. Anethole reduces oxidative stress and improves *in vitro* survival and activation of primordial follicles. Braz J Med Biol Res. (2018) 51:e7129. 10.1590/1414-431x20187129 29846431 PMC5999067

[11] Lonergan P Fair T. Maturation of oocytes *in vitro*. Annu Rev Anim Biosci. (2016) 4:255–68. 10.1146/annurev-animal-022114-110822 26566159

[12] Zhu J Moawad AR Wang CY Li HF Ren JY Dai YF. Advances in *in vitro* production of sheep embryos. Int J Vet Sci Med. (2018) 6:15–26. 10.1016/j.ijvsm.2018.02.003 30761316 PMC6161858

[13] He L He T Farrar S Ji L Liu T Ma X. Antioxidants maintain cellular redox homeostasis by elimination of reactive oxygen species. Cell Physiol Biochem. (2017) 44:532–53. 10.1159/000485089 29145191

[14] Shaban S El-Husseny MWA Abushouk AI Salem AMA Mamdouh M Abdel-Daim MM. Effects of antioxidant supplements on the survival and differentiation of stem cells. Oxid Med Cell Longev. (2017) 2017:5032102. 10.1155/2017/5032102 28770021 PMC5523230

[15] Torres-Ramírez N Ortiz-Hernández R Escobar-Sánchez ML Echeverría-Martínez OM Vázquez-Nin GH. Endoplasmic reticulum stress during mammalian follicular atresia. Endoplasmic Reticulum Intech Open. (2019). 10.5772/intechopen.82687

[16] Yu XX Liu YH Liu XM Wang PC Liu S Miao JK. Ascorbic acid induces global epigenetic reprogramming to promote meiotic maturation and developmental competence of porcine oocytes. Sci Rep. (2018) 8:1–12. 10.1038/s41598-018-24395-y 29666467 PMC5904140

[17] Saeedabadi S Abazari-Kia AH Rajabi H Parivar K Salehi M. Melatonin improves the developmental competence of goat oocytes. Int J Fertil Steril. (2018) 12:157. 10.22074/ijfs.2018.5204 29707934 PMC5936615

[18] Liu M Kun Z Tian-min Xu. The role of BMP15 and GDF9 in the pathogenesis of primary ovarian insufficiency. Hum Fertil. (2019) 24:325–32. 10.1080/14647273.2019.1672107 31607184

[19] Seneda MM Godmann M Murphy BD Kimmins S Bordignon V. Developmental regulation of histone H3 methylation at lysine 4 in the porcine ovary. Reproduction. (2008) 135:829–38. 10.1530/REP-07-0448 18502896

[20] Sha QQ Jiang Y Yu C Xiang Y Dai XX Jiang JC. CFP1-dependent histone H3K4 trimethylation in murine oocytes facilitates ovarian follicle recruitment and ovulation in a cell-nonautonomous manner. Cell Mol Life Sci. (2020) 77:2997–012. 10.1007/s00018-019-03322-y 31676962 PMC11104893

[21] Mateen S Rehman MdT Shahzad S Naeem SS Faizy AF Khand AQ. Antioxidant and anti-inflammatory effects of cinnamaldehyde and eugenol on mononuclear cells of rheumatoid arthritis patients. Eur J Pharmacol. (2019) 852:14–24. 10.1016/j.ejphar.2019.02.031 30796902

[22] Marchese A Barbieri R Coppo E Orhan IE Daglia M Nabavi SF. Antimicrobial activity of eugenol and essential oils containing eugenol: a mechanistic viewpoint. Crit Rev Microbiol. (2017) 43:668–89. 10.1080/1040841X.2017.1295225 28346030

[23] Wie MB Won MH Lee KH Shin JH Lee JC Suh HW. Eugenol protects neuronal cells from excitotoxic and oxidative injury in primary cortical cultures. Neurosci Lett. (1997) 225:93–6. 10.1016/S0304-3940(97)00195-X 9147382

[24] Silva FFM Monte FJQ Lemos TLG Nascimento PGG Medeiros CAK Paiva LMM. Eugenol derivatives: synthesis, characterization, and evaluation of antibacterial and antioxidant activities. Chem Cent J. (2018) 12:1–9. 10.1186/s13065-018-0407-4 29611004 PMC5880794

[25] Vasconcelos EM Costa FC Azevedo AVN Barroso PAA De Assis EIT Paulino LRFM. Eugenol influences the expression of messenger RNAs for superoxide dismutase and glutathione peroxidase 1 in bovine secondary follicles cultured in vitro. Zygote. (2021) 29:301–6. 10.1017/S0967199420000908 33597054

[26] Silva JRV Lucci CM Carvalho FCA Bao SN Costa SHF Santos RR. Effect of coconut water and Braun-Collins solutions at different temperatures and incubation times on the morphology of goat preantral follicles preserved *in vitro*. Theriogenoloy. (2000) 54:809–22. 10.1016/S0093-691X(00)00392-7 11101040

[27] Rodrigues APR Costa SHF Santos RR Amorim CA Lucci CM Báo SN. *In vitro* culture of cryopreserved caprine ovarian tissue pieces and isolated follicles. Cell Preserv Technol. (2006) 4:290–98. 10.1089/cpt.2006.9998

[28] Pfaffl MW. A new mathematical model for relative quantification in real-time RT-PCR. Nucleic Acids Res. (2001) 29:e45. 10.1093/nar/29.9.e45 11328886 PMC55695

[29] Donfack NJ Alves KA Alves BG Rocha RMP Bruno JB Bertolini M. Stroma cell-derived factor 1 and connexins (37 and 43) are preserved after vitrification and in vitro culture of goat ovarian cortex. Theriogenology. (2018) 116:83–8. 10.1016/j.theriogenology.2018.05.001 29783047

[30] Faustino LR Santos RR Silva CMG Pinto LC Celestino JJH Campelo CC. Goat and sheep ovarian tissue cryopreservation: Effects on the morphology and development of primordial follicles and density of stromal cell. Anim Reprod Sci. (2010) 122:90–7. 10.1016/j.anireprosci.2010.08.001 20800393

[31] Ighodaro OM Akinloye AO. First line defence antioxidants-superoxide dismutase (SOD), catalase (CAT) and glutathione peroxidase (GPX): Their fundamental role in the entire antioxidant defence grid. Alex J Med. (2018) 54:287–93. 10.1016/j.ajme.2017.09.001

[32] Kim DY Won KJ Hwang DI Park SM Kim B Lee HM. Chemical composition, antioxidant and anti-melanogenic activities of essential oils from Chrysanthemum boreale Makino at different harvesting stages. Chem Biodivers. (2018) 15:170–06. 10.1002/cbdv.201700506 29292594

[33] Das S Smith TD Sarma JD Ritzenthaler JD Maza J Kaplan BE. ERp29 restricts Connexin43 oligomerization in the endoplasmic reticulum. Mol Biol Cell. (2009) 20:2593–604. 10.1091/mbc.e08-07-0790 19321666 PMC2682600

[34] Han C Liu B Yang T Yang Y Xu J Wei Z. Involvement of PKCε in FSH-induced connexin43 phosphorylation and oocyte maturation in mouse. Biol Open. (2018) 7:bio034678. 10.1242/bio.034678 30061305 PMC6124567

[35] Bus A Szymańska-Vandendriessche K Pintelon I Leroy JLMR Leybaert L Bols PEJ. Preservation of connexin 43 and transzonal projections in isolated bovine pre-antral follicles before and following vitrification. J Assist Reprod Genet. (2021) 38:479–92. 10.1007/s10815-020-01993-2 33159276 PMC7884540

[36] Tokuhiro K Satouh Y Nozawa K Isotani A Fujihara Y Hirashima Y. Calreticulin is required for development of the cumulus oocyte complex and female fertility. Sci Rep. (2015) 5:14254. 10.1038/srep14254 26388295 PMC4585710

[37] Silva JRV Tharasanit T Taverne MAM Van der Weijden GC Santos RR Figueiredo JR. The activin-follistatin system and in vitro early follicle development in goats. J Endocrinol. (2006) 189:113–25. 10.1677/joe.1.06487 16614386

[38] Ahmad HI Liu G Jiang X Edallew SG Wassie T Tesema B. Maximum-likelihood approaches reveal signatures of positive selection in BMP15 and GDF9 genes modulating ovarian function in mammalian female fertility. Ecol Evol. (2017) 7:8895–902. 10.1002/ece3.3336 29177034 PMC5689494

[39] Hui Q Ammeter E Liu S Yang R Lu P Lahaye L. Eugenol attenuates inflammatory response and enhances barrier function during lipopolysaccharide-induced inflammation in the porcine intestinal epithelial cells. J Anim Sci. (2020) 98:245. 10.1093/jas/skaa245 32735667 PMC7531220

[40] Silva AWB Ribeiro RP Menezes VG Barberino RS Passos JRS Dau AMP. Expression of TNF-αsystem members in bovine ovarian follicles and the effects of TNF-α or dexamethasone on preantral follicle survival, development and ultrastructure *in vitro*. Anim Reprod Sci. (2017) 182:56–68. 10.1016/j.anireprosci.2017.04.010 28511863

[41] Manabe N Matsuda-Minehata F Goto Y Maeda A Cheng Y Nakagawa S. Role of cell death ligand and receptor system on regulation of follicular atresia in pig ovaries. Reprod Domest Anim. (2008) 43:268–72. 10.1111/j.1439-0531.2008.01172.x 18638134

[42] Carlberg C Molnár F. The Histone Code. Human Epigenomics. Singapore: Springer (2018).

[43] Howe FS Fischl H Murray SC Mellor J. Is H3K4me3 instructive for transcription activation? Bioessays. (2017) 39:1–12. 10.1002/bies.201600095 28004446

[44] Lu C Yang D Sabbatini ME Aaron H Colby MW Grinstaff NH. Contrasting roles of H3K4me3 and H3K9me3 in regulation of apoptosis and gemcitabine resistance in human pancreatic cancer cells. BMC Cancer. (2018) 18:149. 10.1186/s12885-018-4061-y 29409480 PMC5801751

[45] Yuan L Liu H Liu X Zhang X Wu J Wang Y. Epigenetic modification of H3K4 and oxidative stress are involved in MC-LR-induced apoptosis in testicular cells of SD rats. Environ Toxicol. (2020) 35:277–91. 10.1002/tox.22865 31691492

